# Does a high peritoneal cancer index lead to a worse prognosis of patients with advanced ovarian cancer?: a systematic review and meta-analysis based on the latest evidence

**DOI:** 10.3389/fonc.2024.1421828

**Published:** 2024-07-02

**Authors:** Siyu Wang, Shaoxuan Liu, Fangyuan Liu, Ying Guo, Fengjuan Han

**Affiliations:** ^1^ Department of Obstetrics and Gynecology, Heilongjiang University of Chinese Medicine, Harbin, Heilongjiang, China; ^2^ Department of Obstetrics and Gynecology, First Affiliated Hospital, Heilongjiang University of Chinese Medicine, Harbin, Heilongjiang, China

**Keywords:** peritoneal cancer index, advanced ovarian cancer, cytoreductive surgery, primary debulking surgery, meta-analysis

## Abstract

**Background:**

The newest clinical evidence that the relationship between the peritoneal cancer index (PCI) and the postoperative prognosis of advanced ovarian cancer patients remains controversial, and there are no large-sample and multicenter studies to clarify this matter. Therefore, in this paper, we used meta-analysis to systematically assess the postoperative prognostic value of PCI in subjects with advanced ovarian cancer to provide individualized treatment plans and thus improve the prognosis of patients.

**Methods:**

Literature on the correlation between PCI and the postoperative prognosis in subjects with advanced OC undergoing cytoreductive surgery (CRS) was searched in the Cochrane Library, Pubmed, Embase, and Web of Science from the database inception to April 20, 2023. The search was updated on February 28, 2024. We only included late-stage (FIGO stage: III-IV) patients who did not undergo neoadjuvant chemotherapy (NACT) or hyperthermic intraperitoneal chemotherapy (HIPEC). Afterwards, literature screening and data extraction were conducted using Endnote20 software. The literature quality was assessed using the Newcastle-Ottawa Scale (NOS). Lastly, statistical analysis was performed with STATA 15.0 software.

**Results:**

Five studies with 774 patients were included. The result indicated that patients with high PCI had a worse prognosis than those with low PCI. The combined hazard ratio was 2.79 [95%CI: (2.04, 3.82), p<0.001] for overall survival (OS) in patients with high PCI. Further subgroup analysis by the FIGO staging revealed that in stage III [HR: 2.61, 95%CI: (2.00, 3.40), p<0.001] and stage III-IV patients [HR: 2.69, 95%CI: (1.66, 4.36), p<0.001], a high PCI score was significantly associated with a worse prognosis. The PCI score had a greater impact on the OS of patients with higher stages. The combined hazard ratio was 1.89 [95%CI: (1.51, 2.36), p<0.001] for progression-free survival (PFS) in patients with high PCI.

**Conclusion:**

PCI may be used as a postoperative prognosis indicator in patients with advanced OC on primary debulking surgery. High PCI indicates a worse prognosis. However, further research is warranted to confirm these findings.

**Systematic review registration:**

https://www.crd.york.ac.uk/prospero/, identifier CRD42023424010.

## 1 Introduction

Ovarian cancer (OC) is a highly prevalent malignant tumor within the female reproductive system. Approximately 20,000 cases are diagnosed annually, with 14,000 resulting in fatal outcomes. This disease exhibits the highest mortality rate among gynecological tumors, with a 5-year survival rate of only 40–45% (1, 2). The insidious progression and ease of peritoneal dissemination contribute to approximately 70% of patients being diagnosed at advanced stages, resulting in a dismal prognosis (1). The current standard approach for advanced OC involves cytoreductive surgery (CRS) combined with platinum-taxane chemotherapy. This treatment aims to eliminate all visible lesions and improve the overall prognosis of OC patients (3). However, patients with extensive peritoneal metastases and excessive tumor load face challenges in achieving satisfactory cytoreduction, leading to postoperative residual disease (RD) (4). Consequently, it becomes difficult to provide optimal benefits to patients through the procedure. Neoadjuvant chemotherapy (NACT) is often utilized for individuals who are unsuitable for satisfactory cytoreduction surgery. However, this may prolong the timing of surgery, potentially resulting in disease progression or an increased risk of surgical complications (5). Hence, there is a crucial need for accurate and effective methods to predict patients’ postoperative prognosis. This would allow for the assessment of the feasibility of primary debulking surgery (PDS), ultimately formulating an optimal treatment plan and enhancing patients’ quality of life.

The Peritoneal Cancer Index (PCI) was initially proposed by Sugarbaker et al., and it utilizes a scale that divides the abdomen into 13 zones. Each zone is evaluated and assigned a score from 0 to 3 based on tumor size, resulting in a total score ranging from 0 to 39 (6). The PCI provides an assessment of the size and spread of abdominal tumors, and it has become the standard method for quantifying peritoneal metastasis and guiding treatment decisions. By utilizing the PCI score, it may be possible to evaluate the condition of OC patients and thus guide treatment options. In fact, the PCI has proven to be beneficial in assessing postoperative survival rates in individuals with advanced OC (7–10). Zhou et al (9). found that the PCI can predict whether OC patients are suitable candidates for optimal cytoreductive surgery (OCS). Further research has demonstrated the impact of PCI scores on the postoperative survival prognosis of OC patients. Specifically, patients with a PCI score below 17.5 exhibit better overall survival (OS) and progression-free survival (PFS) outcomes (p < 0.001). In a study by Egger et al. (11), the relationship between PCI and postoperative prognosis in OC patients was investigated. The study revealed that higher PCI scores are associated with worse 5-year OS (p < 0.001) and 5-year disease-free survival (DFS) rates (p < 0.001). Additionally, a recent meta-analysis showcased that higher PCI scores correlate with lower survival rates, indicating a significant negative correlation between PCI and the survival rate of patients with advanced OC (12). However, it is important to note that there are also studies presenting different and controversial findings. For instance, Elzarkaa et al. (13) conducted a prospective study and found no relationship between PCI and patient OS (p=0.716), which aligns with the viewpoint of Engbersen et al. (14), who argue that the PCI score cannot serve as a predictive factor for survival prognosis.

The predictive accuracy of PCI for the prognosis of patients with advanced OC who underwent PDS is currently a subject of controversy. It has not yet been established conclusively. Meta-analysis, a powerful statistical tool, can overcome the limitations posed by different sample sizes in individual studies and generate the most reliable estimates. In light of this, our paper aims to conduct a meta-analysis of existing relevant studies to investigate the correlation between PCI and postoperative prognosis in advanced OC patients. The findings of this study will provide valuable references for clinical applications.

## 2 Methods

The study adhered to the PRISMA guidelines (15), which are the Preferred Reporting Items for Systematic Reviews and Meta-Analyses. The research protocol for this study was registered on PROSPERO (Registration No. CRD42023424010).

### 2.1 Search strategy

A search was conducted in several databases including The Cochrane Library, Pubmed, Embase, and Web of Science to gather relevant literature. The search period was limited to the time between the creation of the database and April 20, 2023. An update to the search was performed on February 28, 2024. The focus of the search was to identify observational studies that examined the correlation between the PCI and the prognosis of individuals with advanced OC who underwent CRS. Subject terms and free-text terms related to ovarian neoplasms, ovarian tumor, cytoreduction surgical procedures”, “cytoreductive surgery”, “peritoneal cancer index”, and “peritoneal carcinomatosis index”. were utilized in the search. To ensure comprehensive results, references in relevant literature were also manually searched. For detailed information regarding the search strategy, please refer to the supplements (**Supplementary Table 1**).

### 2.2 Eligibility

#### 2.2.1 Inclusion criteria

1. The study subjects have advanced OC and have undergone PDS.
2. The outcome indicators were postoperative OS, PFS.
3. PCI was assessed intraoperatively.
4. Observational studies.

#### 2.2.2 Exclusion criteria

1. Reviews, cases, letters, guidelines, theoretical studies, experience summaries, animal experiments, conferences, duplicate publications, etc.
2. PCI scores were obtained through non-surgical approaches.
3. The study population consisted of patients who received NACT or underwent HIPEC.
4. Literature with a sample size of <20.
5. Studies with no explicit outcome indicators and data that could not be extracted.

### 2.3 Data extraction

Two independent investigators, Wang and Liu, conducted literature screening according to the predetermined inclusion and exclusion criteria. Initially, all potentially relevant studies were imported into EndNote 20 to identify and eliminate duplicate studies. Subsequently, a preliminary screening was conducted by evaluating the titles and abstracts to exclude studies that did not meet the criteria. Finally, the full texts of the remaining literature were meticulously reviewed to select studies that met the eligibility criteria. In the event of any disagreements, they were resolved through discussion, and if necessary, a third party (Guo) was consulted to make a final decision.

Using an Excel spreadsheet, Wang and Liu, the two investigators, extracted and documented the following data: the first author’s name, publication date and country, study design, number of subjects, age, International Federation of Gynecology and Obstetrics (FIGO) stage of OC, PCI cut-off value, PCI measuring method, duration of follow-up, and outcome indicators (OS, PFS). In order to address any disputes, Guo, a third party, joins in for discussion.

### 2.4 Quality assessment

Using the Newcastle-Ottawa Scale (NOS) (16), two investigators assessed the literature quality of eligible retrospective studies. The scale comprises three dimensions: selection, comparability, and exposure, with a total of eight items. The NOS score ranges from 0 to 9. Studies that scored 6 or higher were categorized as high-quality, whereas those with a score of 5 or less were classified as low-quality.

### 2.5 Statistical analysis

The data was analyzed using Stata 15.0 as per the instructions. To assess the correlation between PCI and OS and PFS, hazard ratio (HR) and 95% CI were calculated. Quantification of heterogeneity was done through Cochran’s Q test and Higgins I^2^. If the p-value was less than 0.1 or I^2^ was greater than 50%, it indicated statistically significant heterogeneity. In the presence of substantial heterogeneity, the random effects model was used. On the other hand, if low heterogeneity was observed, the fixed effects model was applied. In case of significant heterogeneity, sensitivity analyses and subgroup analyses were conducted to identify the sources of heterogeneity. To visualize publication bias, funnel plots were used when there were more than five literature sources. Furthermore, publication bias was statistically examined using the Egger test. If publication bias was identified, it was addressed using the trim-and-fill method, and the impact on the meta-analysis results was analyzed. A p-value less than 0.05 was considered as indicating a statistically significant difference.

## 3 Results

### 3.1 Search results

**Figure 1:**
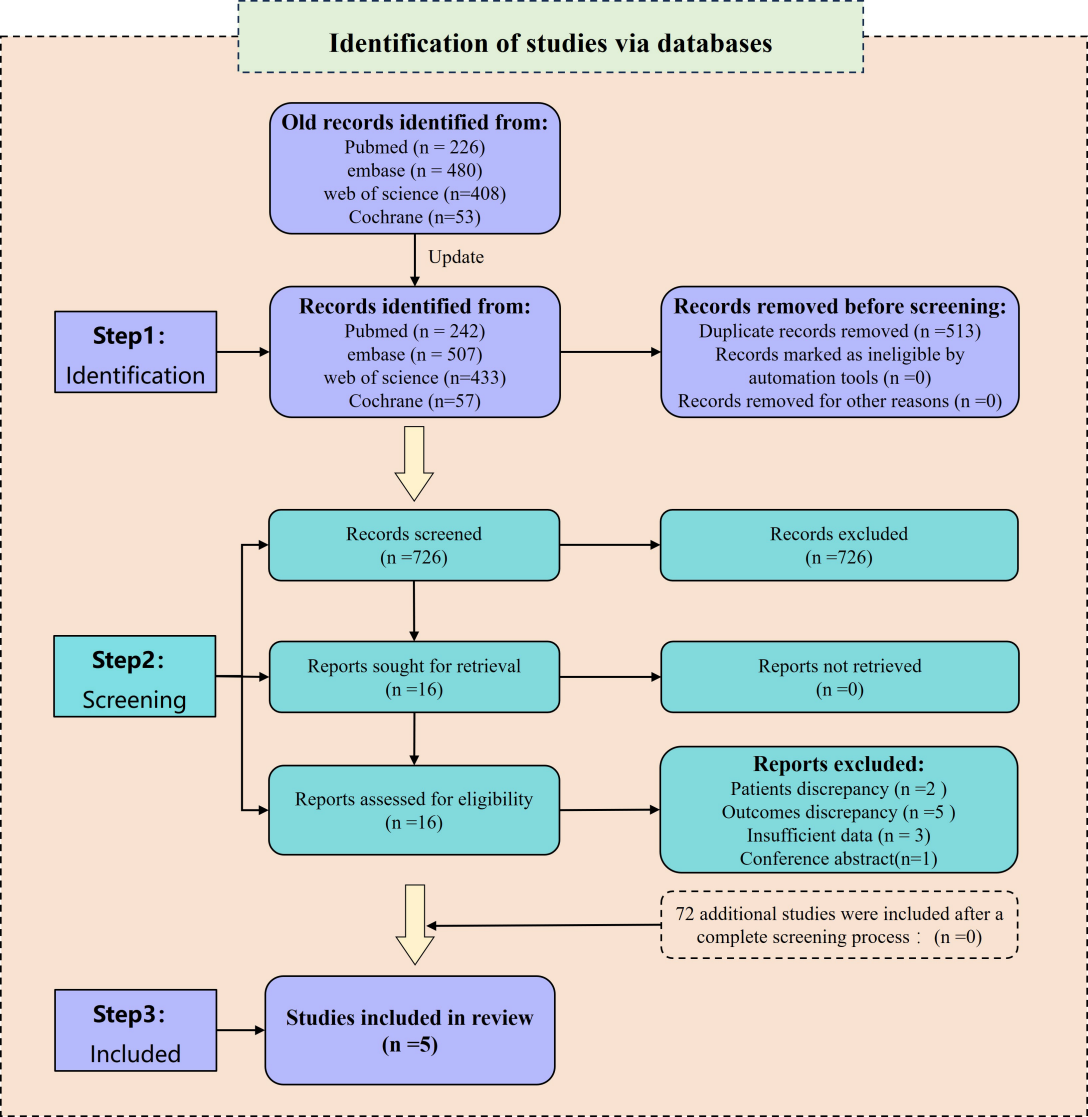
Flow chart showing the process of study screening.

The preliminary search yielded a total of 1,167 relevant reports. Among them, 483 duplicates were excluded using Endnote 20 and manual screening. Furthermore, 668 irrelevant papers were removed after a careful review of their titles and abstracts. After these steps, 16 studies remained for further analysis and their references were investigated. However, among these studies, 5 (7, 17–20) did not provide acceptable outcome indicators, 3 (11, 21, 22) did not include HR values, 1 (23) was a conference abstract, and 2 (24, 25) included patients who had undergone intermittent CRS. Consequently, these 11 papers were excluded as they did not meet the inclusion criteria. Eventually, 5 studies (8–10, 13, 26) were included for the analysis. In the updated search, a total of 1,239 studies were identified. After screening the 72 newly identified studies, none of them met the inclusion criteria. Therefore, no additional studies were included. For a visual representation of the literature screening process, please refer to **Figure 1**.

### 3.2 Characteristics of included studies and quality assessment

Three out of the five studies included in the analysis were retrospective, while the remaining two were prospective in nature. The sample size varied, ranging from 80 to 400 participants, with a total of 774 subjects diagnosed with advanced epithelial OC. All patients underwent PDS after being diagnosed with FIGO stage III-IV disease. However, a portion of the patients experienced RD after the surgery. Following PDS, a total of 770 patients received adjuvant chemotherapy. In one study, the patients were sourced from the Tumor Bank Ovarian Cancer, while the other four studies recruited participants from single-center clinical trials. Laparotomy was conducted in all studies to determine the PCI scores. Based on the PCI cut-off value, the patients were divided into the high PCI group and the low PCI group. The highest PCI cut-off value was 18.5, whereas the lowest cut-off value was 13. All studies examined the association between PCI and OS following PDS in patients with advanced OC. Additionally, two studies investigated the correlation between PCI and PFS. The quality of the included studies was assessed using the NOS, and all studies scored above 6, indicating high quality (**Table 1**).

**Table 1:** Characteristics of studies included in the current meta-analysis.

First Author	Year	The Location of the Study	Patient Source	Study Type	Sample Size	Age (years)	FIGO Stage	OC grading	Histology	PCI determination method	PCI Cut-off Value	Residual Disease	Adjuvant chemotherapy	Follow-up Duration (months)	Outcome Indicators	NOS
K. Gasimli	2015	Germany	Database	Retrospective study	80	58.0^2^	IIIB:8 IIIC:57 IV: 15	G1: 5 G2: 17 G3: 58	Serous: 77 Clear cell: 2 Endometrioid:1	Operative and pathology reports	OS:18 PFS:13	R0:80	Y:76 N:4	29.1^2^	OS PFS	8
A. A. Elzarkaa	2018	Egypt	Single-center	Prospective study	96	52.15^1^	IIIB:33 IIIC:41 IVA: 22	G1/G2: 37 G3: 59	High grade serous:96	Operative	13	R0:62 R1:12 R2:22	Y:96 N:0	24(specified)	OS	8
A. Llueca	2018	Spain	Single-center	Prospective study	80	59.9^1^	IIIC:57 IVA: 23	NK	Serous:44 Others:36	Operative	10	R0:64 R1:6 R2:11	Y:80 N:0	23.2^2^	OS	8
M. Zhou	2020	China	Single-center	Retrospective study	400	<65 339 ≥65 61	IIIC:400	NK	High grade serous:31 Others:369	Operative	17.5	R0/R1: 223 R2:177	Y:400 N:0	85.4^2^	OS PFS	9
M. Asp	2022	Sweden	Single-center	Retrospective study	118	67^1^	III:90 IV: 28	NK	NK	Operative	18.5	R0:75 R1/R2:43	Y:118 N:0	PFS:18.35^2^ OS: 32.91^2^	OS	8

^1^Mean value; ^2^Median value.

PCI, Peritoneal cancer index; OC, Ovarian cancer; NOS, Newcastle-Ottawa Scale; FIGO, International Federation of Gynecology and Obstetrics; R0, Complete cytoreductive surgery (CCS); R1, Optimal cytoreductive surgery (OCS); R2, Suboptimal cytoreductive surgery (SCS); Y, Yes; N, No. NK, Not known.

### 3.3 Outcomes

#### 3.3.1 Correlation between PCI and OS

**Figure 2:**
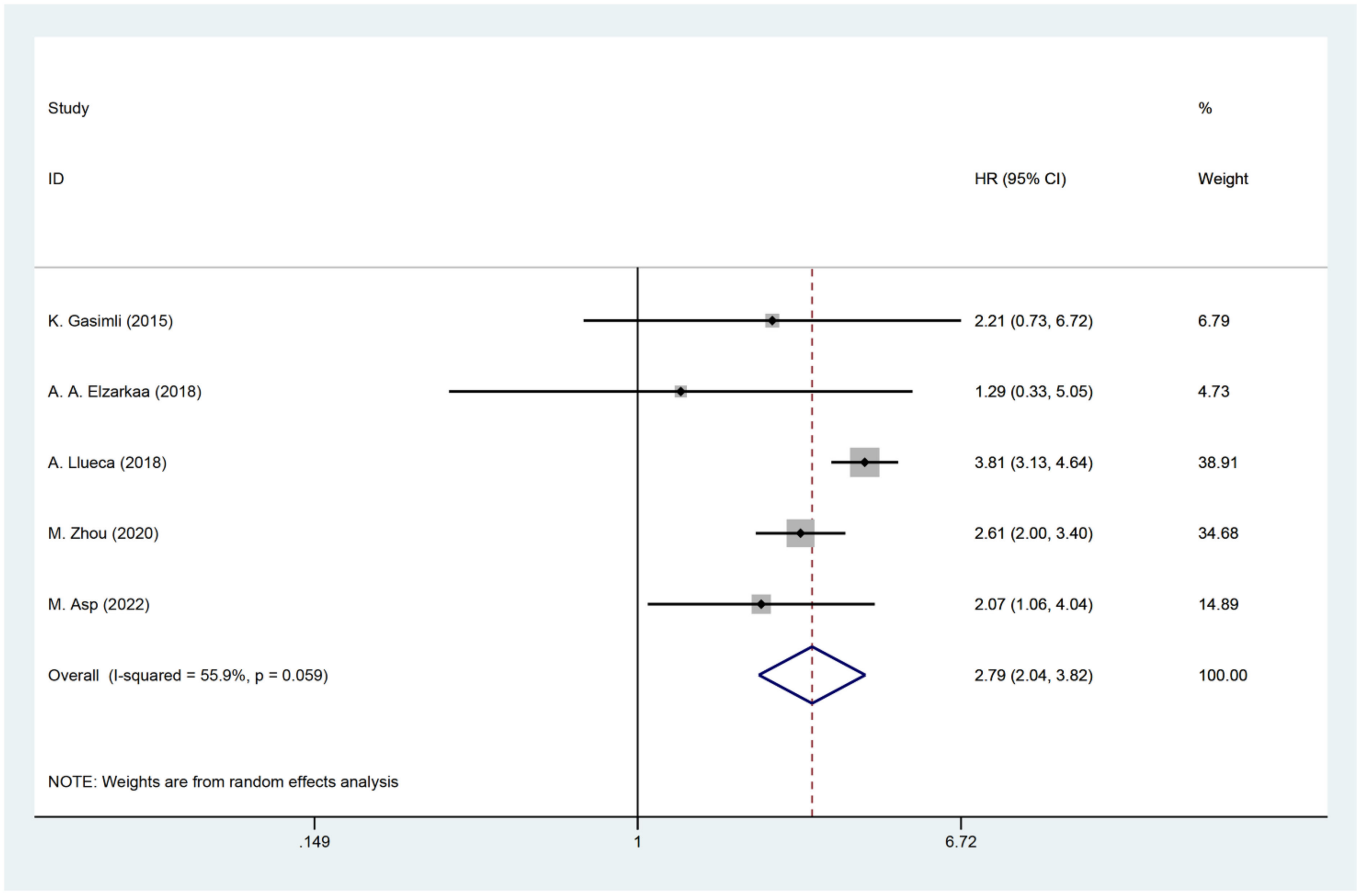
Forest plot for OS analysis.

Five studies have examined the relationship between PCI and postoperative OS in patients with OC. The HR data for OS, obtained from multifactorial analyses, were pooled together. Given the significant heterogeneity observed among the studies (I^2^ = 55.9, P=0.059), a random effects model was employed for the analysis. The results of the meta-analysis demonstrated a significant correlation between PCI and OS [HR=2.79, 95%CI: (2.04, 3.82), *P*<0.001]. These findings suggest that patients with high PCI levels experienced shorter postoperative OS, indicating a worse prognosis compared to those with low PCI levels in OC (**Figure 2**).

**Figure 3:**
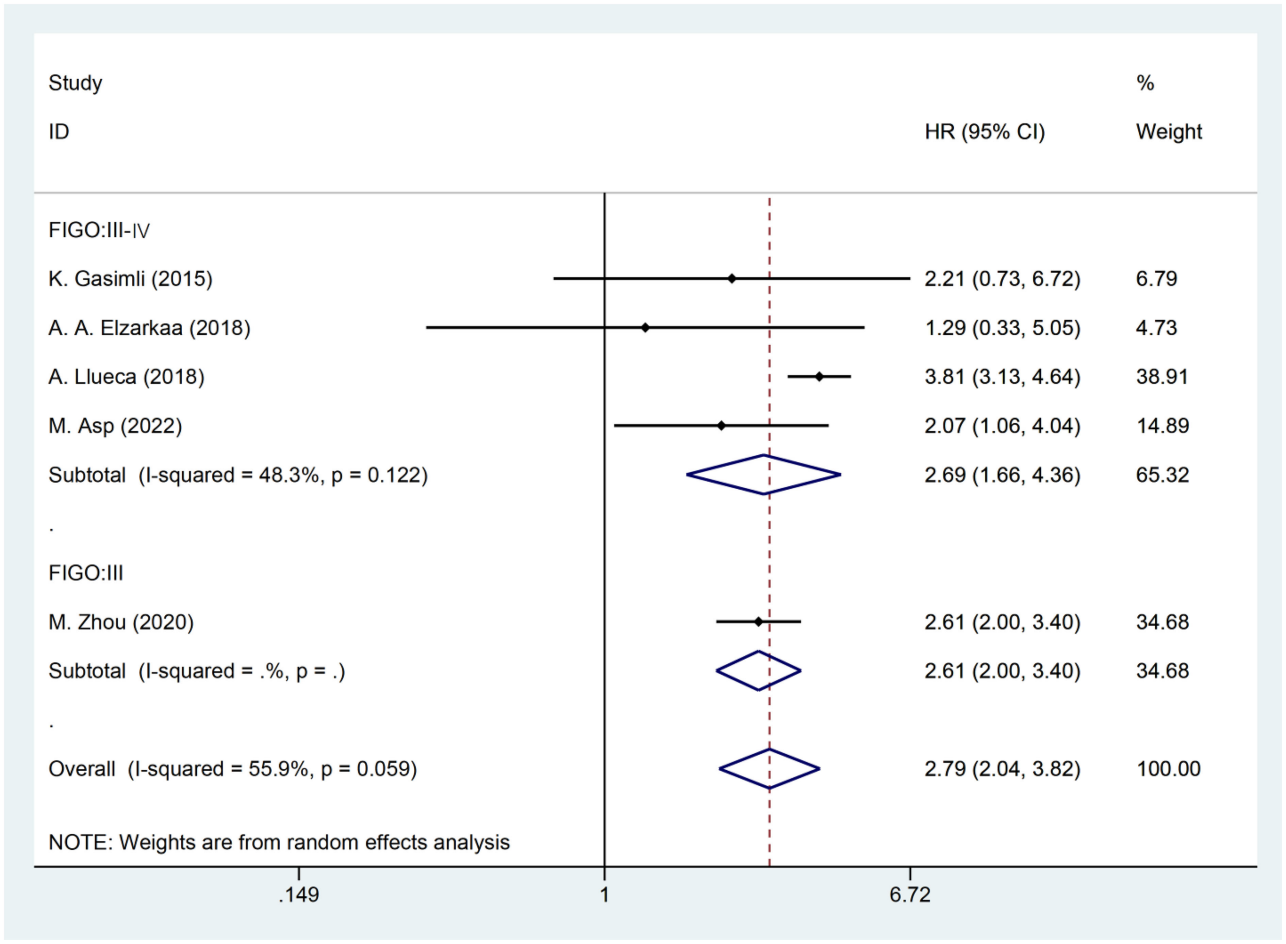
Forest plot for OS subgroup analysis (FIGO stage).

**Figure 4:**
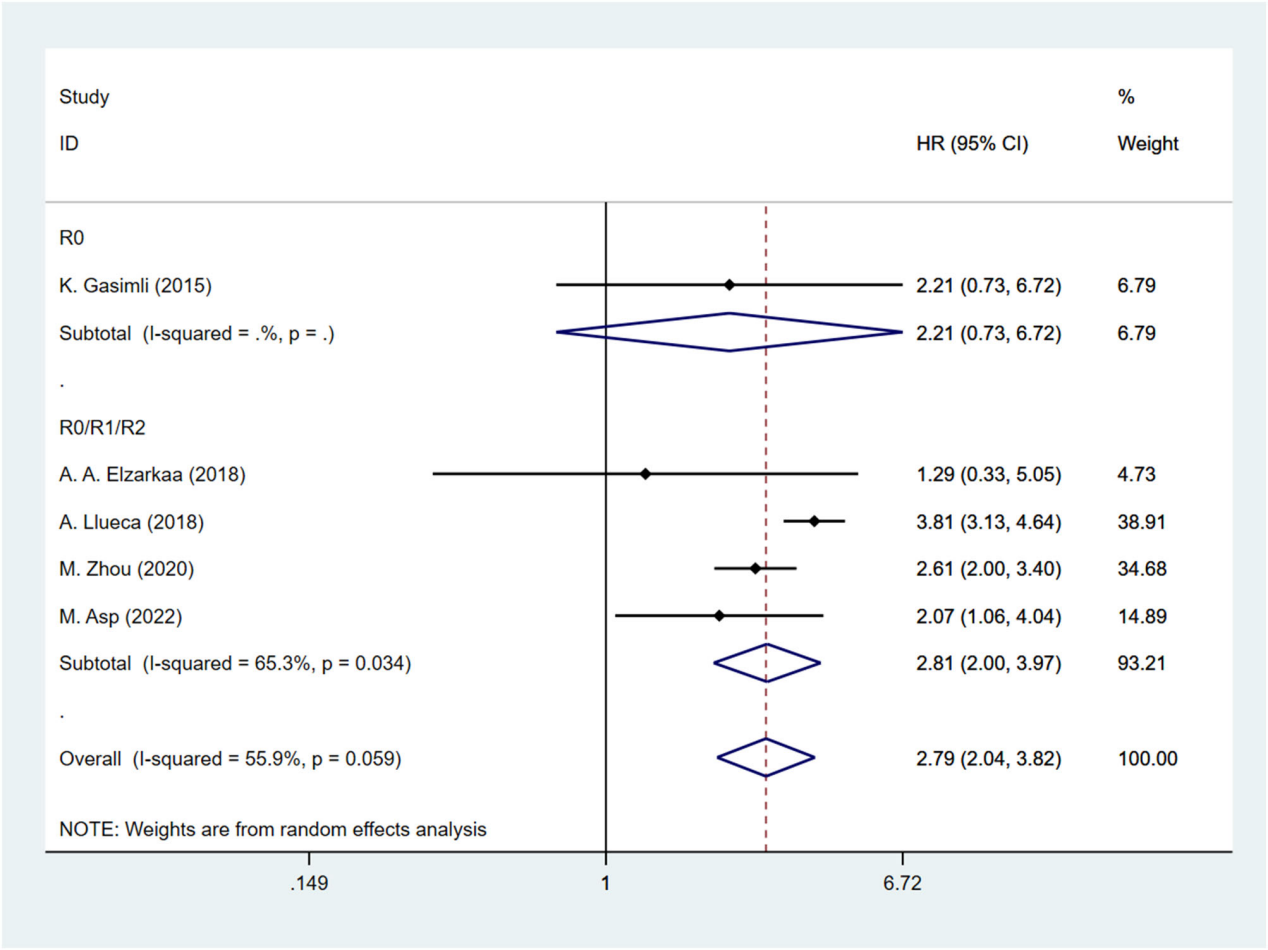
Forest plot for OS subgroup analysis (RD).

The presence of heterogeneity among the studies prompted an investigation into the underlying factors based on the baseline characteristics of the patients. Upon conducting subgroup analysis by the cancer FIGO stage, it was observed that heterogeneity was reduced within the FIGO III-IV group (I^2^ = 48.3, P = 0.122). This finding suggests that the FIGO stage could potentially be a contributing factor to the observed heterogeneity (**Figure 3**). Subgroup analysis by RD was conducted. The results revealed that the presence of residual tumors did not contribute to the heterogeneity observed in the overall analysis (I^2^ = 65.3, P = 0.034). In the studies that reported no residual tumors after surgical intervention, no significant association was found between PCI and OS [HR: 2.21, 95%CI: (0.73–6.72), *P*=0.162]. Even after excluding this particular study, the overall results remained relatively unchanged [HR: 2.81, 95% CI: (2.00–3.97), *P*<0.001] (**Figure 4**).

#### 3.3.2 Correlation between PCI and PFS

**Figure 5:**
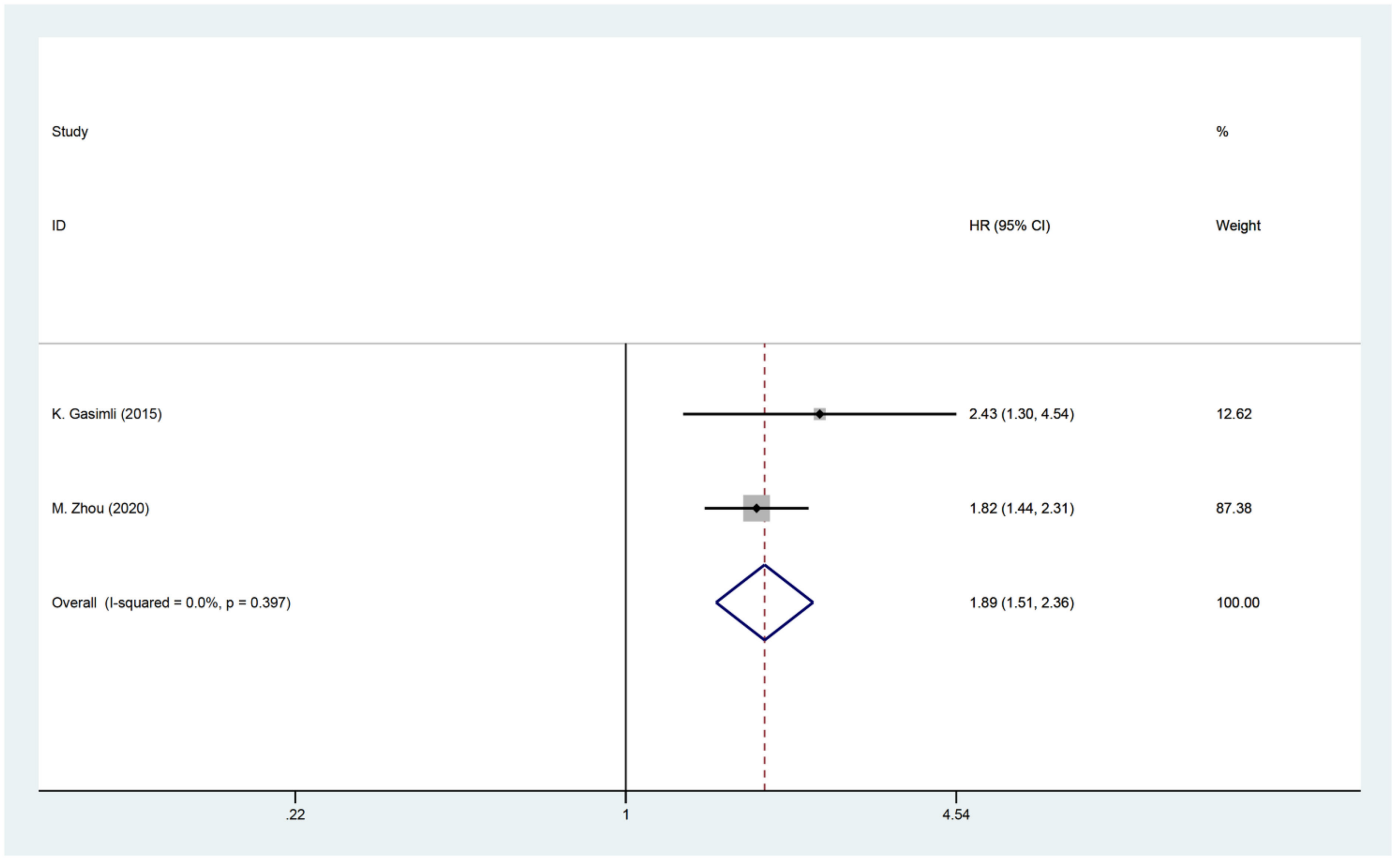
Forest plot for PFS analysis.

Two studies investigated the relationship between PCI and postoperative PFS in patients with OC. The HR data for PFS, obtained from multifactorial analyses, were combined. Since there was minimal heterogeneity among the studies (I^2 ^= 0, P=0.397), a fixed effects model was employed for the analysis. The findings of the meta-analysis revealed a significant correlation between PCI and PFS [HR=1.89, 95% CI: (1.51, 2.36), *P*<0.001]. These results suggest that patients with high PCI experience shorter postoperative PFS and have a worse prognosis compared to OC patients with low PCI (**Figure 5**).

#### 3.3.3 Sensitivity analysis and publication bias

**Figure 6:**
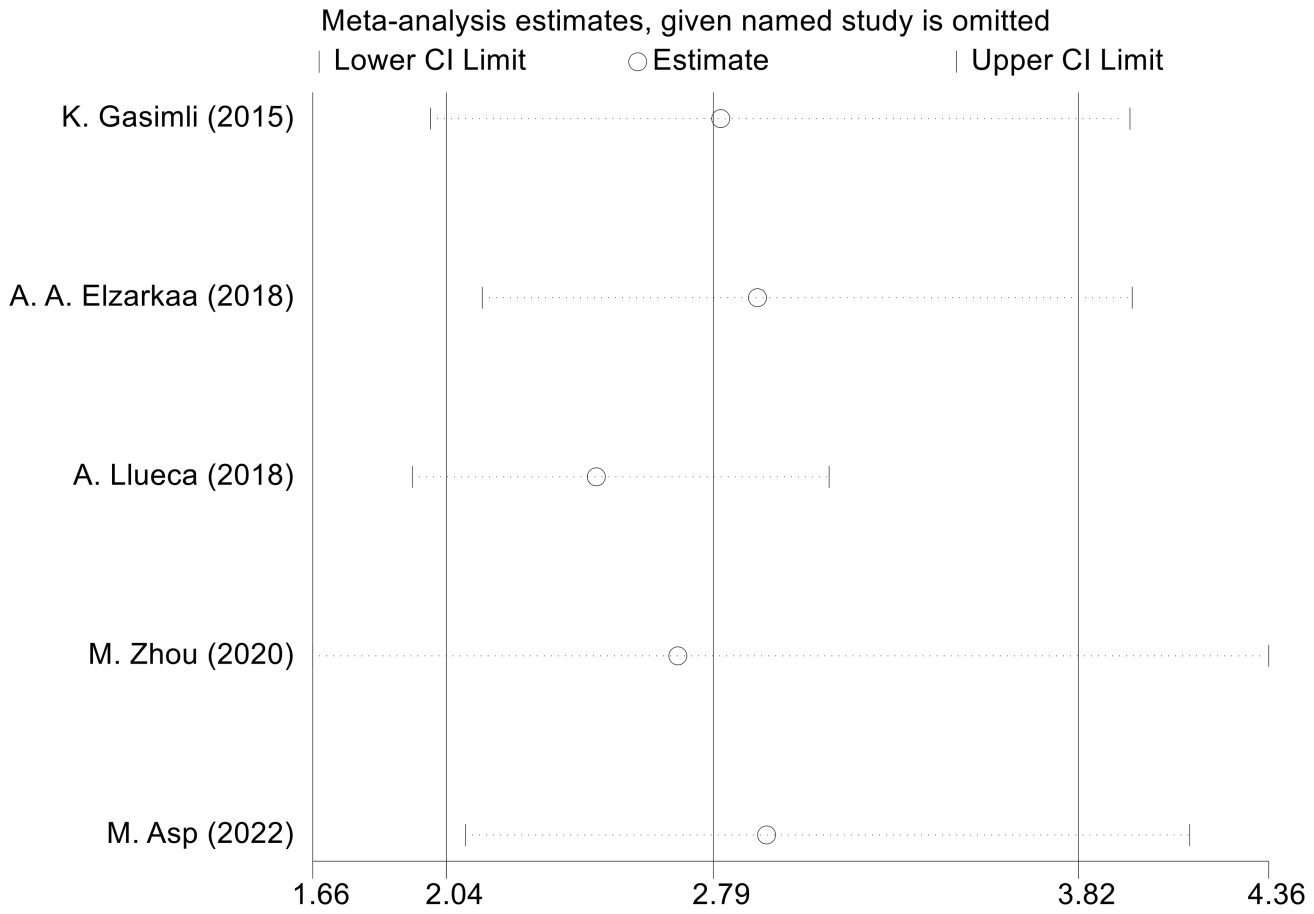
Sensitivity analysis for OS.

We conducted sensitivity analyses by excluding one study at a time. The outcomes revealed that there was no considerable alteration in the combined effect size of OS. This indicates that the findings from the meta-analysis are consistent and robust (**Figure 6**). To assess publication bias regarding OS, we employed the Egger test, which revealed no evidence of publication bias (P=0.153).

## 4 Discussion

This systematic review and meta-analysis assesses the link between primary CRS and postoperative outcomes in advanced OC. Analyzing data from five studies with 774 patients, the research reveals that primary CRS is a significant prognostic indicator for OC patients undergoing PDS. Patients with elevated PCI scores are more likely to experience reduced OS and PFS, underscoring PCI’s utility as a prognostic marker for survival in advanced OC.

The prognostic significance of the PCI for certain malignancies is well recognized, thanks to its ease of use and consistency. A meta-analysis has shown that a high PCI is a predictor of OS in patients with colorectal cancer peritoneal metastases (27). Building on this, Narasimhan et al.’s (28) meta-analysis of eight studies and 1,279 patients confirmed that an increase in PCI raises the mortality risk by 10.25% for those who underwent CRS and HIPEC [HR 1.10, 95% CI: (1.05, 1.15)]. Additionally, a recent study found that gastric cancer patients with advanced peritoneal metastases and a PCI of 12 or less had a more favorable prognosis when treated with S-1 and oxaliplatin (SOX) combined with intraperitoneal high-dose paclitaxel, achieving a median OS of 22.7 months, compared to only 15.0 months for those with a PCI above 12 [HR = 2.24; 95% CI:(1.13, 4.43)] (29). These findings underscore the predictive power of PCI for OS in cancer patients. PCI is a robust stratification tool, offering insights into tumor volume and distribution, which are crucial for gauging the extent of peritoneal spread. Considering the propensity of advanced OC for peritoneal metastasis, PCI is a promising prognostic indicator for OC patients. Our meta-analysis reinforces the utility of PCI in evaluating postoperative survival outcomes in advanced OC, with the study corroborating PCI’s predictive accuracy for both OS and PFS, consistent with findings across different cancer types.

Our study, in tandem with a meta-analysis (12), has firmly established a link between the PCI and poor prognosis in OC patients. Yang et al. (12) detailed survival rates across various PCI thresholds, showing significantly lower five-year survival for those exceeding the threshold. To bolster the robustness of our findings, we included recent and contentious prospective clinical studies, applying rigorous inclusion and exclusion criteria. By conducting a distinct analysis of PCI’s impact on OS and PFS, we further reinforced PCI’s prognostic significance in postoperative advanced OC patients undergoing PDS. Clinical studies suggest that NACT and HIPEC may alter the postoperative prognosis of advanced cancer patients (5, 30). Although these treatments can enhance CRS success rates and improve patients’ quality of life (31), their efficacy is still debated (32, 33). Therefore, we focused on studies involving patients who received PDS and adjuvant chemotherapy, deliberately excluding those treated with NACT or HIPEC, which led to less heterogeneity in our statistical results. Further subgroup analysis by FIGO stage revealed that PCI’s predictive power is more pronounced in patients with higher FIGO stages undergoing PDS, underscoring its potential for prognostic assessment in advanced OC. We also pinpointed potential sources of heterogeneity. Despite the modest number of studies included, our research provides substantial evidence supporting PCI’s role in postoperative prognosis prediction for advanced OC patients post-PDS, offering a valuable and contemporary reference for future clinical application.

The FIGO staging system is pivotal for assessing the prognosis of OC patients, with higher stages correlating with increased tumor spread and reduced survival rates. However, the FIGO stage alone might not fully capture the tumor burden and its distribution throughout the abdominal cavity. The depth and location of metastases in advanced OC can complicate surgery, and lower FIGO stages do not necessarily imply less invasive surgery or better outcomes for patients with peritoneal metastasis. Fortunately, the PCI offers a more nuanced assessment, evaluating not just the extent of peritoneal metastasis but also the tumor’s precise distribution and volume (21). Our study’s findings, along with a cohort study, emphasize the importance of PCI in predicting survival prognosis. High PCI values have been linked to adverse responses to CRS and chemotherapy, with a PCI score above 11 indicating a poorer prognosis (7) and significantly lower postoperative survival rates in advanced OC patients (P < 0.0253) (21). This suggests that a high PCI score is detrimental to OC patient survival, aligning with the consensus from numerous studies.

CRS is the cornerstone of treatment for advanced OC, with maximum tumor reduction closely linked to improved median survival times (34). A recent network meta-analysis has underscored the prognostic impact of RD in OC patients undergoing PDS, revealing that RD of less than 1 cm is tied to reduced OS compared to no RD present [HR=2; 95% CI: (1.8, 2.2)] (4). The PCI has proven to be a reliable predictor for achieving optimal cytoreduction in advanced OC. Studies indicate that a PCI score above 20 confers a significantly heightened risk of RD, nearly nine times greater (p=0.003) (18). This suggests that patients with elevated PCI scores are more likely to face challenges in completely removing tumors surgically, increasing the likelihood of postoperative RD. Jónsdóttir et al. proposed that a PCI exceeding 24 might prompt consideration of NACT to shrink tumor volume and facilitate optimal cytoreduction (17). Consequently, a high PCI is a vital prognostic indicator for evaluating the feasibility of tumor resection in advanced OC (19).

Laparoscopy is the gold standard for assessing the PCI score, offering precision and reliability. However, in advanced OC, early surgical assessment of PCI is crucial to decide between PDS or NACT, thus preventing unnecessary invasive procedures (35). Research indicates that CT-based PCI scoring is an effective tool for quantifying peritoneal disease and predicting the 5-year survival rate in advanced OC patients (36). Moreover, a strong correlation exists between CT-based and surgical PCI scores (37, 38). Consequently, preoperative PCI evaluation through imaging like CT or MRI offers a practical, accurate, and non-invasive alternative. The adoption of Minimally invasive surgery (MIS) in gynecologic oncology is on the rise, offering significant benefits. Gallotta et al.’s study (39) demonstrated that elderly and geriatric patients with gynecologic tumors experienced favorable short-term outcomes with a minimally invasive robotic technique. Moreover, a comprehensive systematic review and meta-analysis suggest that MIS could supplant traditional laparotomy in determining the viability of tumor removal for advanced OC patients. Experienced surgeons utilizing MIS have also been shown to enhance outcomes following laparoscopic pelvic and PDS (40), underscoring the growing importance of MIS in improving patient care and surgical precision.

Laparoscopy stands as a notable advancement in MIS and is increasingly recognized for its reliability in predicting the outcomes of PDS in OC patients before treatment. Fagotti et al. have devised the Fagotti score model, leveraging laparoscopy to forecast the feasibility of OCS. This model uses the PIV to gauge patient satisfaction following PDS (41). Staging laparoscopy (S-LPS) allows for the PIV assessment at various stages, offering a thorough analysis of the patient’s prognosis. Vizzielli et al. expanded on this by examining the PIV before, during, and after surgery with S-LPS. They refined the laparoscopic scoring system into the Vizzielli score, which incorporates factors such as physical health, ascites presence, and serum CA 125 levels to estimate the risk of postoperative complications in OC patients. Patients with a Vizzielli score between 6 and 8 are identified to be at high risk, with complication rates as high as 37.1% (42). Moreover, S-LPS is instrumental in evaluating peritoneal metastasis in advanced epithelial OC, with a PIV score above 8 indicating a higher risk of RD post-PDS and poorer OS and PFS (43). Thus, S-LPS is pivotal in assessing the prognosis of advanced OC patients post-PDS.

Currently, interval debulking surgery post-NACT is an established alternative for patients facing PDS when complete tumor excision is unfeasible (41). To evaluate OS and PFS after PDS, and to gauge the appropriateness of NACT, factors such as the PCI, Fagotti score, and Vizzielli score are meticulously assessed and evaluated. Evidence suggests that the Fagotti score retains its predictive value for tumor excision extent even post-NACT, with its variations pre- and post-NACT serving as prognostic indicators for OS and PFS in advanced OC (44). Laparoscopic evaluation using the Fagotti score is a crucial tool for foretelling surgical results, irrespective of NACT administration. Gueli Alletti et al. (45) propose that patients in complete clinical remission after NACT are candidates for laparoscopic interval debulking surgery. This approach not only enhances quality of life over conventional laparotomy but also maintains PFS, offering a viable and less invasive treatment strategy for these patients. The PCI serves as a multifaceted tool for physicians, providing significant value across several domains. It assists in assessing the viability of PDS, crafting efficacious treatment plans, pinpointing patients who may benefit from NACT, and bolstering the success rates of CRS. Additionally, PCI aids in reducing postoperative complications and substantially improves the overall quality of life for patients (24, 46). Assessing the feasibility of PDS in advanced OC patients and predicting survival outcomes are significantly important. To achieve this, the evaluation of PCI using non-invasive imaging techniques, in addition to the calculation of Fagotti score and Vizzielli score by MIS, plays a crucial role. The obtained information is valuable for developing personalized treatment plans.

This study represents an initial foray into investigating the potential of PCI as a postoperative prognostic indicator for advanced OC patients undergoing PDS. By prioritizing rigorous studies and conducting multifactorial analyses, the presented results possess substantial reliability and value. However, it is important to acknowledge certain limitations. Firstly, the study included a limited number of literature references and outcome indicators, with a relatively small sample size of patients. These factors may potentially impact the accuracy and applicability of our findings. Additionally, the lack of standardized cut-off values for PCI across the included studies, influenced by individual patient conditions, poses an additional challenge. As such, while this study provides evidence supporting the predictive capability of PCI in evaluating postoperative prognosis for OC patients, further subgroup analyses are necessary to establish the optimal cut-off value for PCI. It is crucial to acknowledge that various factors, such as RD and BRCA status, specific chemotherapy regimens, and maintenance therapy, have a significant impact on the OS of patients. However, it is important to note that this study’s multivariate analysis did not consider these specific factors. Consequently, the focus of this study was to elucidate the effects of primary CRS on the OS and PFS of advanced OC patients based on available data. To fully comprehend the prognostic implications for patients with advanced OC following PDS, conducting larger prospective studies that incorporate these key factors is imperative. Furthermore, establishing a defined cut-off value for PCI and assessing patients’ PCI prior to surgery are crucial. By doing so, precise treatment protocols can be developed for patients with high PCI.

## 5 Conclusion

This meta-analysis, which includes five studies, indicates a negative correlation between higher PCI and survival outcomes (OS and PFS) in advanced OC patients after PDS. The significance of this association is particularly pronounced in patients with higher FIGO stages. However, it is important to note that the existing clinical studies and evidence have certain limitations. Therefore, further studies with a larger sample size, longer follow-up duration, and more rigorous design are needed to validate these findings.

## Data availability statement

The original contributions presented in the study are included in the article/**Supplementary Material**. Further inquiries can be directed to the corresponding author.

## Author contributions

SW: Formal analysis, Methodology, Writing – original draft, Writing – review & editing, Investigation. SL: Formal analysis, Investigation, Methodology, Writing – original draft. FL: Conceptualization, Formal analysis, Methodology, Writing – original draft. YG: Conceptualization, Writing – original draft. FH: Funding acquisition, Resources, Supervision, Formal analysis, Writing – original draft.
