# Supplementary material for: Does a high peritoneal cancer index lead to a worse prognosis of patients with advanced ovarian cancer?: a systematic review and meta-analysis based on the latest evidence
Source: Front Oncol. 2024 Jul 2;14:1421828. doi: 10.3389/fonc.2024.1421828 (PMC11249540; doi:10.3389/fonc.2024.1421828)
Supplement: Supplementary file 1 [file Table_1.docx]

**Supplementary materials**

**Table S1** Search strategy

1. PubMed

| Search number | Query | Search Details | Results |
| --- | --- | --- | --- |
| 11 | (((("Ovarian Neoplasms"[Mesh]) OR ((((((((((((((((((((((((((((((ovarian neoplasms[Title/Abstract]) OR (ovarian neoplasm[Title/Abstract])) OR (ovary neoplasms[Title/Abstract])) OR (ovary neoplasm[Title/Abstract])) OR (ovary cancer[Title/Abstract])) OR (ovary cancers[Title/Abstract])) OR (ovarian cancer[Title/Abstract])) OR (ovarian cancers[Title/Abstract])) OR (cancer of ovary[Title/Abstract])) OR (cancer of the ovary[Title/Abstract])) OR (ovarian malignance[Title/Abstract])) OR (oophoroma[Title/Abstract])) OR (ovary tumor[Title/Abstract])) OR (neoplasm of the ovary[Title/Abstract])) OR (neoplasms of the ovary[Title/Abstract])) OR (neoplastic ovarian[Title/Abstract])) OR (neoplastic ovaries[Title/Abstract])) OR (neoplastic ovary[Title/Abstract])) OR (ovarian neoplasia[Title/Abstract])) OR (ovarian tumor[Title/Abstract])) OR (ovarian tumorigenesis[Title/Abstract])) OR (ovarian tumour[Title/Abstract])) OR (ovarium tumor[Title/Abstract])) OR (ovarium tumour[Title/Abstract])) OR (ovary tumorigenesis[Title/Abstract])) OR (ovary tumour[Title/Abstract])) OR (tumor of the ovary[Title/Abstract])) OR (tumors of the ovary[Title/Abstract])) OR (tumour of the ovary[Title/Abstract])) OR (tumours of the ovary[Title/Abstract]))) OR ((((ovarian[Title/Abstract]) OR (ovary[Title/Abstract])) OR (ovarium[Title/Abstract])) AND ((((((((neoplasms[Title/Abstract]) OR (neoplasm[Title/Abstract])) OR (cancer[Title/Abstract])) OR (cancers[Title/Abstract])) OR (tumor[Title/Abstract])) OR (tumors[Title/Abstract])) OR (tumour[Title/Abstract])) OR (tumours[Title/Abstract])))) AND (("Cytoreduction Surgical Procedures"[Mesh]) OR (((((((((((((cytoreduction surgical procedures[Title/Abstract]) OR (cytoreduction surgical procedure[Title/Abstract])) OR (debulking surgical procedures[Title/Abstract])) OR (debulking surgical procedure[Title/Abstract])) OR (cytoreductive surgeries[Title/Abstract])) OR (cytoreductive surgery[Title/Abstract])) OR (cytoreductive surgical procedures[Title/Abstract])) OR (cytoreductive surgical procedure[Title/Abstract])) OR (debulking procedure[Title/Abstract])) OR (debulking procedures[Title/Abstract])) OR (debulking surgery[Title/Abstract])) OR (debulking surgeries[Title/Abstract])) OR (cytoreduction[Title/Abstract])))) AND ((peritoneal cancer index[Title/Abstract]) OR (peritoneal carcinomatosis index[Title/Abstract])) | ("Ovarian Neoplasms"[MeSH Terms] OR ("Ovarian Neoplasms"[Title/Abstract] OR "ovarian neoplasm"[Title/Abstract] OR "ovary neoplasms"[Title/Abstract] OR "ovary neoplasm"[Title/Abstract] OR "ovary cancer"[Title/Abstract] OR "ovary cancers"[Title/Abstract] OR "ovarian cancer"[Title/Abstract] OR "ovarian cancers"[Title/Abstract] OR "cancer of ovary"[Title/Abstract] OR "cancer of the ovary"[Title/Abstract] OR "ovarian malignance"[Title/Abstract] OR "oophoroma"[Title/Abstract] OR "ovary tumor"[Title/Abstract] OR "neoplasm of the ovary"[Title/Abstract] OR "neoplasms of the ovary"[Title/Abstract] OR "neoplastic ovarian"[Title/Abstract] OR "neoplastic ovaries"[Title/Abstract] OR "neoplastic ovary"[Title/Abstract] OR "ovarian neoplasia"[Title/Abstract] OR "ovarian tumor"[Title/Abstract] OR "ovarian tumorigenesis"[Title/Abstract] OR "ovarian tumour"[Title/Abstract] OR "ovarium tumor"[Title/Abstract] OR (("ovary"[MeSH Terms] OR "ovary"[All Fields] OR "ovarium"[All Fields]) AND "tumour"[Title/Abstract]) OR "ovary tumorigenesis"[Title/Abstract] OR "ovary tumour"[Title/Abstract] OR "tumor of the ovary"[Title/Abstract] OR "tumors of the ovary"[Title/Abstract] OR "tumour of the ovary"[Title/Abstract] OR "tumours of the ovary"[Title/Abstract]) OR (("ovarian"[Title/Abstract] OR "ovary"[Title/Abstract] OR "ovarium"[Title/Abstract]) AND ("neoplasms"[Title/Abstract] OR "neoplasm"[Title/Abstract] OR "cancer"[Title/Abstract] OR "cancers"[Title/Abstract] OR "tumor"[Title/Abstract] OR "tumors"[Title/Abstract] OR "tumour"[Title/Abstract] OR "tumours"[Title/Abstract]))) AND ("Cytoreduction Surgical Procedures"[MeSH Terms] OR ("Cytoreduction Surgical Procedures"[Title/Abstract] OR "cytoreduction surgical procedure"[Title/Abstract] OR "debulking surgical procedures"[Title/Abstract] OR "debulking surgical procedure"[Title/Abstract] OR "cytoreductive surgeries"[Title/Abstract] OR "cytoreductive surgery"[Title/Abstract] OR "cytoreductive surgical procedures"[Title/Abstract] OR "cytoreductive surgical procedure"[Title/Abstract] OR "debulking procedure"[Title/Abstract] OR "debulking procedures"[Title/Abstract] OR "debulking surgery"[Title/Abstract] OR "debulking surgeries"[Title/Abstract] OR "cytoreduction"[Title/Abstract])) AND ("peritoneal cancer index"[Title/Abstract] OR "peritoneal carcinomatosis index"[Title/Abstract]) | 242 |
| 10 | (peritoneal cancer index[Title/Abstract]) OR (peritoneal carcinomatosis index[Title/Abstract]) | "peritoneal cancer index"[Title/Abstract] OR "peritoneal carcinomatosis index"[Title/Abstract] | 1068 |
| 9 | ("Cytoreduction Surgical Procedures"[Mesh]) OR (((((((((((((cytoreduction surgical procedures[Title/Abstract]) OR (cytoreduction surgical procedure[Title/Abstract])) OR (debulking surgical procedures[Title/Abstract])) OR (debulking surgical procedure[Title/Abstract])) OR (cytoreductive surgeries[Title/Abstract])) OR (cytoreductive surgery[Title/Abstract])) OR (cytoreductive surgical procedures[Title/Abstract])) OR (cytoreductive surgical procedure[Title/Abstract])) OR (debulking procedure[Title/Abstract])) OR (debulking procedures[Title/Abstract])) OR (debulking surgery[Title/Abstract])) OR (debulking surgeries[Title/Abstract])) OR (cytoreduction[Title/Abstract])) | "Cytoreduction Surgical Procedures"[MeSH Terms] OR "Cytoreduction Surgical Procedures"[Title/Abstract] OR "cytoreduction surgical procedure"[Title/Abstract] OR "debulking surgical procedures"[Title/Abstract] OR "debulking surgical procedure"[Title/Abstract] OR "cytoreductive surgeries"[Title/Abstract] OR "cytoreductive surgery"[Title/Abstract] OR "cytoreductive surgical procedures"[Title/Abstract] OR "cytoreductive surgical procedure"[Title/Abstract] OR "debulking procedure"[Title/Abstract] OR "debulking procedures"[Title/Abstract] OR "debulking surgery"[Title/Abstract] OR "debulking surgeries"[Title/Abstract] OR "cytoreduction"[Title/Abstract] | 13,737 |
| 8 | ((((((((((((cytoreduction surgical procedures[Title/Abstract]) OR (cytoreduction surgical procedure[Title/Abstract])) OR (debulking surgical procedures[Title/Abstract])) OR (debulking surgical procedure[Title/Abstract])) OR (cytoreductive surgeries[Title/Abstract])) OR (cytoreductive surgery[Title/Abstract])) OR (cytoreductive surgical procedures[Title/Abstract])) OR (cytoreductive surgical procedure[Title/Abstract])) OR (debulking procedure[Title/Abstract])) OR (debulking procedures[Title/Abstract])) OR (debulking surgery[Title/Abstract])) OR (debulking surgeries[Title/Abstract])) OR (cytoreduction[Title/Abstract]) | "cytoreduction surgical procedures"[Title/Abstract] OR "cytoreduction surgical procedure"[Title/Abstract] OR "debulking surgical procedures"[Title/Abstract] OR "debulking surgical procedure"[Title/Abstract] OR "cytoreductive surgeries"[Title/Abstract] OR "cytoreductive surgery"[Title/Abstract] OR "cytoreductive surgical procedures"[Title/Abstract] OR "cytoreductive surgical procedure"[Title/Abstract] OR "debulking procedure"[Title/Abstract] OR "debulking procedures"[Title/Abstract] OR "debulking surgery"[Title/Abstract] OR "debulking surgeries"[Title/Abstract] OR "cytoreduction"[Title/Abstract] | 12,625 |
| 7 | "Cytoreduction Surgical Procedures"[Mesh] | "Cytoreduction Surgical Procedures"[MeSH Terms] | 4,216 |
| 6 | (("Ovarian Neoplasms"[Mesh]) OR ((((((((((((((((((((((((((((((ovarian neoplasms[Title/Abstract]) OR (ovarian neoplasm[Title/Abstract])) OR (ovary neoplasms[Title/Abstract])) OR (ovary neoplasm[Title/Abstract])) OR (ovary cancer[Title/Abstract])) OR (ovary cancers[Title/Abstract])) OR (ovarian cancer[Title/Abstract])) OR (ovarian cancers[Title/Abstract])) OR (cancer of ovary[Title/Abstract])) OR (cancer of the ovary[Title/Abstract])) OR (ovarian malignance[Title/Abstract])) OR (oophoroma[Title/Abstract])) OR (ovary tumor[Title/Abstract])) OR (neoplasm of the ovary[Title/Abstract])) OR (neoplasms of the ovary[Title/Abstract])) OR (neoplastic ovarian[Title/Abstract])) OR (neoplastic ovaries[Title/Abstract])) OR (neoplastic ovary[Title/Abstract])) OR (ovarian neoplasia[Title/Abstract])) OR (ovarian tumor[Title/Abstract])) OR (ovarian tumorigenesis[Title/Abstract])) OR (ovarian tumour[Title/Abstract])) OR (ovarium tumor[Title/Abstract])) OR (ovarium tumour[Title/Abstract])) OR (ovary tumorigenesis[Title/Abstract])) OR (ovary tumour[Title/Abstract])) OR (tumor of the ovary[Title/Abstract])) OR (tumors of the ovary[Title/Abstract])) OR (tumour of the ovary[Title/Abstract])) OR (tumours of the ovary[Title/Abstract]))) OR ((((ovarian[Title/Abstract]) OR (ovary[Title/Abstract])) OR (ovarium[Title/Abstract])) AND ((((((((neoplasms[Title/Abstract]) OR (neoplasm[Title/Abstract])) OR (cancer[Title/Abstract])) OR (cancers[Title/Abstract])) OR (tumor[Title/Abstract])) OR (tumors[Title/Abstract])) OR (tumour[Title/Abstract])) OR (tumours[Title/Abstract]))) | "Ovarian Neoplasms"[MeSH Terms] OR ("Ovarian Neoplasms"[Title/Abstract] OR "ovarian neoplasm"[Title/Abstract] OR "ovary neoplasms"[Title/Abstract] OR "ovary neoplasm"[Title/Abstract] OR "ovary cancer"[Title/Abstract] OR "ovary cancers"[Title/Abstract] OR "ovarian cancer"[Title/Abstract] OR "ovarian cancers"[Title/Abstract] OR "cancer of ovary"[Title/Abstract] OR "cancer of the ovary"[Title/Abstract] OR "ovarian malignance"[Title/Abstract] OR "oophoroma"[Title/Abstract] OR "ovary tumor"[Title/Abstract] OR "neoplasm of the ovary"[Title/Abstract] OR "neoplasms of the ovary"[Title/Abstract] OR "neoplastic ovarian"[Title/Abstract] OR "neoplastic ovaries"[Title/Abstract] OR "neoplastic ovary"[Title/Abstract] OR "ovarian neoplasia"[Title/Abstract] OR "ovarian tumor"[Title/Abstract] OR "ovarian tumorigenesis"[Title/Abstract] OR "ovarian tumour"[Title/Abstract] OR "ovarium tumor"[Title/Abstract] OR (("ovary"[MeSH Terms] OR "ovary"[All Fields] OR "ovarium"[All Fields]) AND "tumour"[Title/Abstract]) OR "ovary tumorigenesis"[Title/Abstract] OR "ovary tumour"[Title/Abstract] OR "tumor of the ovary"[Title/Abstract] OR "tumors of the ovary"[Title/Abstract] OR "tumour of the ovary"[Title/Abstract] OR "tumours of the ovary"[Title/Abstract]) OR (("ovarian"[Title/Abstract] OR "ovary"[Title/Abstract] OR "ovarium"[Title/Abstract]) AND ("neoplasms"[Title/Abstract] OR "neoplasm"[Title/Abstract] OR "cancer"[Title/Abstract] OR "cancers"[Title/Abstract] OR "tumor"[Title/Abstract] OR "tumors"[Title/Abstract] OR "tumour"[Title/Abstract] OR "tumours"[Title/Abstract])) | 150,526 |
| 5 | (((ovarian[Title/Abstract]) OR (ovary[Title/Abstract])) OR (ovarium[Title/Abstract])) AND ((((((((neoplasms[Title/Abstract]) OR (neoplasm[Title/Abstract])) OR (cancer[Title/Abstract])) OR (cancers[Title/Abstract])) OR (tumor[Title/Abstract])) OR (tumors[Title/Abstract])) OR (tumour[Title/Abstract])) OR (tumours[Title/Abstract])) | ("ovarian"[Title/Abstract] OR "ovary"[Title/Abstract] OR "ovarium"[Title/Abstract]) AND ("neoplasms"[Title/Abstract] OR "neoplasm"[Title/Abstract] OR "cancer"[Title/Abstract] OR "cancers"[Title/Abstract] OR "tumor"[Title/Abstract] OR "tumors"[Title/Abstract] OR "tumour"[Title/Abstract] OR "tumours"[Title/Abstract]) | 126,439 |
| 4 | (((((((neoplasms[Title/Abstract]) OR (neoplasm[Title/Abstract])) OR (cancer[Title/Abstract])) OR (cancers[Title/Abstract])) OR (tumor[Title/Abstract])) OR (tumors[Title/Abstract])) OR (tumour[Title/Abstract])) OR (tumours[Title/Abstract]) | "neoplasms"[Title/Abstract] OR "neoplasm"[Title/Abstract] OR "cancer"[Title/Abstract] OR "cancers"[Title/Abstract] OR "tumor"[Title/Abstract] OR "tumors"[Title/Abstract] OR "tumour"[Title/Abstract] OR "tumours"[Title/Abstract] | 3,652,276 |
| 3 | ((ovarian[Title/Abstract]) OR (ovary[Title/Abstract])) OR (ovarium[Title/Abstract]) | "ovarian"[Title/Abstract] OR "ovary"[Title/Abstract] OR "ovarium"[Title/Abstract] | 271,827 |
| 2 | (((((((((((((((((((((((((((((ovarian neoplasms[Title/Abstract]) OR (ovarian neoplasm[Title/Abstract])) OR (ovary neoplasms[Title/Abstract])) OR (ovary neoplasm[Title/Abstract])) OR (ovary cancer[Title/Abstract])) OR (ovary cancers[Title/Abstract])) OR (ovarian cancer[Title/Abstract])) OR (ovarian cancers[Title/Abstract])) OR (cancer of ovary[Title/Abstract])) OR (cancer of the ovary[Title/Abstract])) OR (ovarian malignance[Title/Abstract])) OR (oophoroma[Title/Abstract])) OR (ovary tumor[Title/Abstract])) OR (neoplasm of the ovary[Title/Abstract])) OR (neoplasms of the ovary[Title/Abstract])) OR (neoplastic ovarian[Title/Abstract])) OR (neoplastic ovaries[Title/Abstract])) OR (neoplastic ovary[Title/Abstract])) OR (ovarian neoplasia[Title/Abstract])) OR (ovarian tumor[Title/Abstract])) OR (ovarian tumorigenesis[Title/Abstract])) OR (ovarian tumour[Title/Abstract])) OR (ovarium tumor[Title/Abstract])) OR (ovarium tumour[Title/Abstract])) OR (ovary tumorigenesis[Title/Abstract])) OR (ovary tumour[Title/Abstract])) OR (tumor of the ovary[Title/Abstract])) OR (tumors of the ovary[Title/Abstract])) OR (tumour of the ovary[Title/Abstract])) OR (tumours of the ovary[Title/Abstract]) | "ovarian neoplasms"[Title/Abstract] OR "ovarian neoplasm"[Title/Abstract] OR "ovary neoplasms"[Title/Abstract] OR "ovary neoplasm"[Title/Abstract] OR "ovary cancer"[Title/Abstract] OR "ovary cancers"[Title/Abstract] OR "ovarian cancer"[Title/Abstract] OR "ovarian cancers"[Title/Abstract] OR "cancer of ovary"[Title/Abstract] OR "cancer of the ovary"[Title/Abstract] OR "ovarian malignance"[Title/Abstract] OR "oophoroma"[Title/Abstract] OR "ovary tumor"[Title/Abstract] OR "neoplasm of the ovary"[Title/Abstract] OR "neoplasms of the ovary"[Title/Abstract] OR "neoplastic ovarian"[Title/Abstract] OR "neoplastic ovaries"[Title/Abstract] OR "neoplastic ovary"[Title/Abstract] OR "ovarian neoplasia"[Title/Abstract] OR "ovarian tumor"[Title/Abstract] OR "ovarian tumorigenesis"[Title/Abstract] OR "ovarian tumour"[Title/Abstract] OR "ovarium tumor"[Title/Abstract] OR (("ovary"[MeSH Terms] OR "ovary"[All Fields] OR "ovarium"[All Fields]) AND "tumour"[Title/Abstract]) OR "ovary tumorigenesis"[Title/Abstract] OR "ovary tumour"[Title/Abstract] OR "tumor of the ovary"[Title/Abstract] OR "tumors of the ovary"[Title/Abstract] OR "tumour of the ovary"[Title/Abstract] OR "tumours of the ovary"[Title/Abstract] | 84,178 |
| 1 | "Ovarian Neoplasms"[Mesh] | "Ovarian Neoplasms"[MeSH Terms] | 97,369 |

2. Embase

| No. | Query | Results |
| --- | --- | --- |
| #11 | #6 AND #9 AND #10 | 507 |
| #10 | 'peritoneal cancer index':ti,ab,kw OR 'peritoneal carcinomatosis index':ti,ab,kw | 1855 |
| #9 | #7 OR #8 | 30509 |
| #8 | 'cytoreductive surgery':ti,ab,kw OR 'cytoreduction surgical procedures':ti,ab,kw OR 'cytoreduction surgical procedure':ti,ab,kw OR 'debulking surgical procedures':ti,ab,kw OR 'debulking surgical procedure':ti,ab,kw OR 'cytoreductive surgeries':ti,ab,kw OR 'cytoreductive surgical procedures':ti,ab,kw OR 'cytoreductive surgical procedure':ti,ab,kw OR cytoreduction:ti,ab,kw OR 'debulking procedure':ti,ab,kw OR 'debulking procedures':ti,ab,kw OR 'debulking surgery':ti,ab,kw OR 'debulking surgeries':ti,ab,kw | 21155 |
| #7 | 'cytoreductive surgery'/exp | 23133 |
| #6 | #1 OR #2 OR #5 | 245379 |
| #5 | #3 AND #4 | 184668 |
| #4 | neoplasm:ti,ab,kw OR neoplasms:ti,ab,kw OR cancer:ti,ab,kw OR cancers:ti,ab,kw OR tumor:ti,ab,kw OR tumors:ti,ab,kw OR tumour:ti,ab,kw OR tumours:ti,ab,kw | 5005786 |
| #3 | ovarian:ti,ab,kw OR ovary:ti,ab,kw OR ovarium:ti,ab,kw | 368646 |
| #2 | 'ovarian neoplasms':ti,ab,kw OR 'ovarian neoplasm':ti,ab,kw OR 'ovary neoplasms':ti,ab,kw OR 'ovary neoplasm':ti,ab,kw OR 'ovary cancer':ti,ab,kw OR 'ovary cancers':ti,ab,kw OR 'ovarian cancer':ti,ab,kw OR 'ovarian cancers':ti,ab,kw OR 'cancer of ovary':ti,ab,kw OR 'cancer of the ovary':ti,ab,kw OR 'ovarian malignance':ti,ab,kw OR oophoroma:ti,ab,kw OR 'ovary tumor':ti,ab,kw OR 'neoplasm of the ovary':ti,ab,kw OR 'neoplasms of the ovary':ti,ab,kw OR 'neoplastic ovarian':ti,ab,kw OR 'neoplastic ovaries':ti,ab,kw OR 'neoplastic ovary':ti,ab,kw OR 'ovarian neoplasia':ti,ab,kw OR 'ovarian tumor':ti,ab,kw OR 'ovarian tumorigenesis':ti,ab,kw OR 'ovarian tumour':ti,ab,kw OR 'ovarium tumor':ti,ab,kw OR 'ovarium tumour':ti,ab,kw OR 'ovary tumorigenesis':ti,ab,kw OR 'ovary tumour':ti,ab,kw OR 'tumor of the ovary':ti,ab,kw OR 'tumors of the ovary':ti,ab,kw OR 'tumour of the ovary':ti,ab,kw OR 'tumours of the ovary':ti,ab,kw | 122448 |
| #1 | 'ovary tumor'/exp | 194612 |

3. Web of Science

| No. | Query | Results |
| --- | --- | --- |
| #1 | ovarian neoplasms (Topic) or ovarian neoplasm (Topic) or ovary neoplasms (Topic) or ovary neoplasm (Topic) or ovary cancers (Topic) or ovary cancer (Topic) or ovarian cancer (Topic) or ovarian cancers (Topic) or cancer of ovary (Topic) or cancer of the ovary (Topic) or ovarian malignance (Topic) or oophoroma (Topic) or ovary tumor (Topic) or neoplasm of the ovary (Topic) or neoplasms of the ovary (Topic) or neoplastic ovarian (Topic) or neoplastic ovaries (Topic) or neoplastic ovary (Topic) or ovarian neoplasia (Topic) or ovarian tumor (Topic) or ovarian tumorigenesis (Topic) or ovarian tumour (Topic) or ovarium tumor (Topic) or ovarium tumour (Topic) or ovary tumorigenesis (Topic) or ovary tumour (Topic) or tumor of the ovary (Topic) or tumors of the ovary (Topic) or tumour of the ovary (Topic) or tumours of the ovary (Topic) | 170176 |
| #2 | ovarian (Topic) or ovary (Topic) or ovarium (Topic) | 350564 |
| #3 | neoplasms (Topic) or neoplasm (Topic) or cancer (Topic) or cancers (Topic) or tumor (Topic) or tumors (Topic) or tumour (Topic) or tumours (Topic) | 4338324 |
| #4 | #2 AND #3 | 169614 |
| #5 | #1 OR #4 | 170180 |
| #6 | cytoreduction surgical procedures (Topic) or cytoreduction surgical procedure (Topic) or debulking surgical procedures (Topic) or debulking surgical procedure (Topic) or cytoreductive surgeries (Topic) or cytoreductive surgical procedures (Topic) or cytoreductive surgical procedure (Topic) or cytoreduction (Topic) or cytoreductive surgery (Topic) or debulking procedure (Topic) or debulking procedures (Topic) or debulking surgery (Topic) or debulking surgeries (Topic) | 17752 |
| #7 | peritoneal cancer index (Topic) or peritoneal carcinomatosis index (Topic) | 2035 |
| #8 | #5 AND #6 AND #7 | 433 |

4. Cochrane

| ID | Search | Hits |
| --- | --- | --- |
| #1 | MeSH descriptor: [Ovarian Neoplasms] explode all trees | 3428 |
| #2 | (ovarian neoplasms):ti,ab,kw OR (ovarian neoplasm):ti,ab,kw OR (ovary neoplasms):ti,ab,kw OR (ovary neoplasm):ti,ab,kw OR (ovary cancer):ti,ab,kw | 7148 |
| #3 | (ovary cancers):ti,ab,kw OR (ovarian cancer):ti,ab,kw OR (ovarian cancers):ti,ab,kw OR (cancer of ovary):ti,ab,kw OR (cancer of the ovary):ti,ab,kw | 9111 |
| #4 | (ovarian malignance):ti,ab,kw OR (oophoroma):ti,ab,kw OR (ovary tumor):ti,ab,kw OR (neoplasm of the ovary):ti,ab,kw OR (neoplasms of the ovary):ti,ab,kw | 2782 |
| #5 | (neoplastic ovarian):ti,ab,kw OR (neoplastic ovaries):ti,ab,kw OR (neoplastic ovary):ti,ab,kw OR (ovarian neoplasia):ti,ab,kw OR (ovarian tumor):ti,ab,kw | 2853 |
| #6 | (ovarian tumorigenesis):ti,ab,kw OR (ovarian tumour):ti,ab,kw OR (ovarium tumor):ti,ab,kw OR (ovarium tumour):ti,ab,kw OR (ovary tumorigenesis):ti,ab,kw | 2777 |
| #7 | (ovary tumour):ti,ab,kw OR (tumor of the ovary):ti,ab,kw OR (tumors of the ovary):ti,ab,kw OR (tumour of the ovary):ti,ab,kw OR (tumours of the ovary):ti,ab,kw | 2004 |
| #8 | #2 or #3 or #4 or #5 or #6 or #7 | 9943 |
| #9 | (ovarian):ti,ab,kw OR (ovary):ti,ab,kw OR (ovarium):ti,ab,kw | 22671 |
| #10 | (neoplasms):ti,ab,kw OR (neoplasm):ti,ab,kw OR (cancer):ti,ab,kw OR (cancers):ti,ab,kw OR (tumor):ti,ab,kw | 254080 |
| #11 | (tumors):ti,ab,kw OR (tumour):ti,ab,kw OR (tumours):ti,ab,kw | 92418 |
| #12 | #10 or #11 | 256572 |
| #13 | #9 and #12 | 9962 |
| #14 | #1 or #8 or #13 | 10047 |
| #15 | MeSH descriptor: [Cytoreduction Surgical Procedures] explode all trees | 310 |
| #16 | (cytoreduction surgical procedures):ti,ab,kw OR (cytoreduction surgical procedure):ti,ab,kw OR (debulking surgical procedures):ti,ab,kw OR (debulking surgical procedure):ti,ab,kw OR (cytoreductive surgeries):ti,ab,kw | 436 |
| #17 | (cytoreductive surgical procedures):ti,ab,kw OR (cytoreductive surgical procedure):ti,ab,kw OR (cytoreduction):ti,ab,kw OR (cytoreductive surgery):ti,ab,kw OR (debulking procedure):ti,ab,kw | 1658 |
| #18 | (debulking procedures):ti,ab,kw OR (debulking surgery):ti,ab,kw OR (debulking surgeries):ti,ab,kw | 761 |
| #19 | #15 or #16 or #17 or #18 | 1974 |
| #20 | (peritoneal cancer index):ti,ab,kw OR (peritoneal carcinomatosis index):ti,ab,kw | 232 |
| #21 | #14 and #19 and #20 | 57 |
